# Knowledge on and use of the FORTA (“Fit fOR The Aged”)-list and the FORTA App by general practitioners in Baden-Württemberg, Germany

**DOI:** 10.1007/s41999-020-00311-4

**Published:** 2020-03-17

**Authors:** Liesbeth Meyer, Martin Wehling

**Affiliations:** Institute of Clinical Pharmacology, Medical Faculty Mannheim, Ruprecht-Karls-University Heidelberg, Theodor-Kutzer-Ufer 1-3, 68167 Mannheim, Germany

**Keywords:** FORTA, Geriatric medicine, Survey, Polypharmacy, Medication tools

## Abstract

**Purpose:**

Drug treatment of older people is complicated as multimorbidity and polypharmacy are highly prevalent. To increase the drug appropriateness in older people, the FORTA (“Fit fOR The Aged”)-list was developed. A survey was designed to describe knowledge and use of the FORTA list and the related FORTA App in a German state.

**Methods:**

Survey conducted among 872 general practitioners (GP) from districts (*n* = 694; 79.6%) or from district-free cities (*n* = 178; 20.4%) in Baden-Württemberg, Germany who were contacted by e-mail and/or phone.

**Results:**

The response rate was 9.4%. 48% of GP claim to know the FORTA list, 54.5% the FORTA App. 32.5% use one of them regularly, 27% at least once a week.

**Conclusions:**

Responding GP expose knowledge and use of the FORTA list and/or the App exceeding expectations given the limited time since the wider appearance of either tool. These results, though limited by a low response rate, tentatively indicate their successful dissemination underlining utility, implementability and teachability.

## Key summary points

**Aim:**

To identify the knowledge on and use of the FORTA (“Fit fOR The Aged”)-list and FORTA App in Baden-Württemberg, Germany.

**Findings:**

48% of responding GP claim to know the FORTA list, 54.5% of them the FORTA App. 32.5% use at least one of them regularly, 27% at least once a week.

**Message:**

The results may indicate that the medication tools the FORTA list, and the FORTA App has been successfully disseminated underlining their utility, implementability and teachability though the response rate was low (9.4%).

## Introduction

In 2060, older adults (65 +) are expected to comprise almost 24% of the population in the USA [1]. With more than 20% of the residents aged 60 and above in 2017, the ageing process is most advanced in Northern America and Europe [2].

To guide physicians in their efforts to rapidly optimize and prioritize medications on the basis of benefit, risk and appropriateness in older patients, the FORTA (Fit fOR The Aged) classification was introduced in 2008 [3]. FORTA represents the first classification system that combines both negative (harmful or critical drugs, D and C labels) and positive (beneficial drugs, A and B labels) labelling at the level of individual drug or drug groups. The system and the derived FORTA list [4], last updated in 2018 [5], now contains 296 medications or medication groups corresponding to 30 indications relevant for older people. The related FORTA App for all major smartphone systems was first provided in 2017. The FORTA list is among the few positive–negative lists [6] that have been clinically validated in a randomized controlled trial showing improvement in drug side effects and patients’ well-being (Barthel-index) [7].

So far, little is known about the dissemination and use of the FORTA list or App in clinical practice though—in addition to scientific papers—intense continuous medical education (CME) activities and a book on drugs for the aged [8] were launched to support this. Here we report on a survey on the knowledge and use of the tools by general practitioners (GP) in the German state of Baden-Württemberg.

## Methods

Using an online search engine [9], GP were identified in the German state of Baden-Württemberg. Inclusion criteria were (1) GP with (2) a published e-mail address for a single doctor or group practices. According to the database entries, GP in districts were separated from those in district-free cities.

In total 872 GP, 694 GP in the districts and 178 GP in district-free cities of Baden-Württemberg were identified and contacted. In a first round of an e-mail-based survey in 2018, 27 of 872 doctors (3.1%) replied. Due to the low response rate, a second round of the e-mail-based survey followed by a subsequent telephone survey (if no response was received) was started in 2019. The results of the telephone survey (appr. duration 5 min) were documented and participants received a protocol of the survey by e-mail.

The survey included seven short questions with no more than three options to answer (tick boxes). The optional eighth question asked for further comments (narrative) on the FORTA list or App (Table 1).

**Table 1:** Questionnaire of the survey

Question	Possible answers
Do you know the FORTA (“Fit fOR The Aged”)-list?	Yes
	No
If “yes”, how often do you use the FORTA list?	Daily
	1–2 times a week
	Less than 1–2 times a week
Do you know the FORTA (“Fit fOR The Aged”) App?	Yes
	No
Do you use the FORTA App?	Yes
	No
If “yes”, how often do you use the FORTA App?	Daily
	1–2 times a week
	Less than 1–2 times a week
Does FORTA help you with the setting/assessment of your patient’s medication?	Yes
	No
How satisfied are you with the FORTA list or the FORTA App?	Very satisfied
	Fair
	Not satisfied
Field for comments	

The participants were assured that the survey was completely anonymous and was to be used only for research purposes. The survey was a quality improvement project without patient interventions. It was approved by the local officer for data.

## Results

The survey was completed by 84 (9.7%) of 872 GP in Baden-Württemberg.

**Fig. 1:**
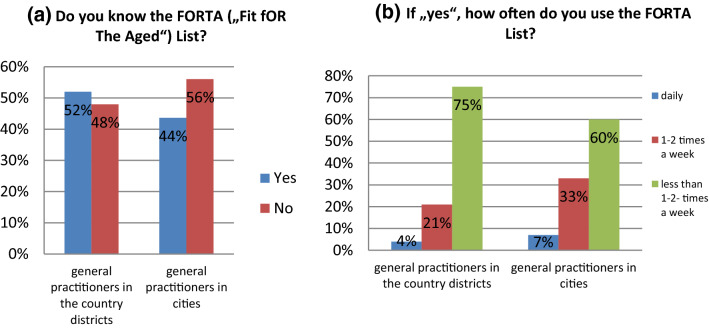
**a** Responses of GP in Baden-Württemberg on their knowledge of the FORTA list for country districts and district-free cities, **b** frequency of using the FORTA list by GP in Baden-Württemberg for country districts and district-free cities

The first question (“Do you know the FORTA (“Fit fOR The Aged”)-list?”) was positively answered by 52% (*n* = 24) of GP in the districts and 44% (*n* = 15) of GP in the district-free cities. Those G responding negatively did not have to answer more questions. The results are shown in Fig. 1a.

Subsequently asked on the frequency of using the FORTA list, the majority of the participants, 75% of GP in the country districts and 60% of GP in the cities confirmed to use the FORTA list, but less than once or twice times per week. Daily use was reported to be below 10% (4% of the general practitioners in the country districts and 7% of the cities practitioners). 21% of GP in the country districts and 33% of GP in the cities reported the use of FORTA once to twice per week (Fig. 1b).

**Fig. 2:**
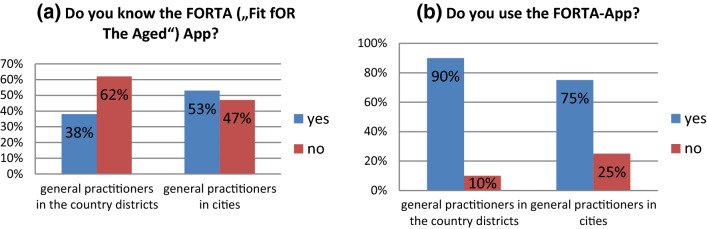
**a** Responses of GP in Baden-Württemberg on their knowledge of the FORTA App for country districts and district-free cities, **b** users or non-users of the FORTA App for country districts and district-free cities

Furthermore, GP were asked whether to know the FORTA App and if they use the app. 38% (*n* = 10) of GP in the country districts and 53% (*n* = 8) of GP in the cities reported to know the FORTA App and most of them declared to use the app: 90% (*n* = 9) of GP in the country districts and 75% (*n* = 6) of GP in the cities (Fig. 2a/b).

**Fig. 3:**
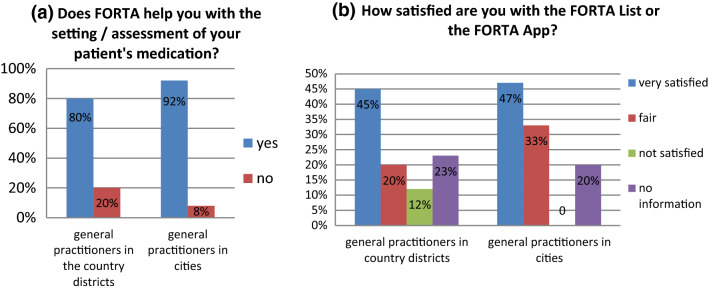
**a** Is FORTA useful for the medication process of GP in Baden-Württemberg for country districts and district-free cities? **b** Degree of satisfaction with FORTA: very satisfied, fair or not satisfied for GP in Baden-Württemberg in country districts and district-free cities

The next question asked whether FORTA is helpful to GP for the setting/assessment of a patient’s medication. The majority [80% (*n* = 19) of in the country districts and 92% (*n* = 12) of GP in the cities] of GP confirmed its helpfulness (Fig. 3a).

Finally, GP were able to choose from three options to define how satisfied they are with the use of FORTA: “Very satisfied”, “fair” or “not satisfied”. Almost half of GP were very satisfied with FORTA [45% (*n* = 13) of GP in the country districts and 47% (*n* = 7) of GP in the cities], 20% (*n* = 5) of GP in the country districts and 33% (*n* = 5) of GP in the cities rated it with fair, 12% (*n* = 3) of GP in the country districts and 0% (*n* = 0) of GP in the cities were not satisfied (Fig. 3b).

Feedback from GP in country districts versus district-free cities did not appear to be systematically or statistically (chi-square test) different.

## Discussion

The problem of multimorbidity leading to polypharmacy in older patients has been addressed—among other strategies—by various drug listing approaches (for review see [6]). Though drug review processes have been generally acknowledged to be essential for the well-being of older people [10], details on their structure, validity, implementability and general utility are sparse. This also applies to the listing approaches that have been created in large numbers, but only rarely validated in clinical trials. For those 73 listing approaches found in a recent systematic review [6], only 13 clinical validation studies could be identified; even these few studies, however, pointed to a clinical superiority of patient-in-focus-listing approaches (PILA) such as the FORTA list or the Screening Tool to Alert Doctors to the Right Treatment (START)/Screening Tool of Older Persons’ Prescriptions (STOPP) criteria [11] over drug-oriented listing approaches (DOLA) such as the Beers list [12] or the European Union (EU)(7)-PIM list [13]. In general, PILA comprise positive and negative recommendations; DOLA are mainly PIM (potentially inappropriate medications)-lists compiling negative recommendations.

With those clinical limitations already obvious at the listing level, even listing approaches with positive clinical validation have to pass yet another hurdle to become relevant to medical care: its dissemination to clinical practice. Even for one of the best studied listing approaches, the START/STOPP criteria [11], no study can be identified in Medline if the word “dissemination” is added to “START/STOPP”. This transition from studies to practice that may be called secondary translation should be as intensively addressed as the clinical characterization of a listing instrument.

To our pleasant surprise, this survey confirmed a dissemination of the FORTA list and/or App to about half of GP responding to this survey. It was found to be clinically very satisfactory also by about half of GP using it.

Though a strict causality cannot be proven, the dissemination strategy appears to have been at least partially successful that resulted in this profound penetration of the tools. This strategy comprised lectures by the FORTA specialists (about one hundred by the last author per year), the publishing of a book specifically addressing drug therapy in older people [8] and the creation of an App easing the access to the FORTA list. By personal observation, latter measure was probably the most influential one. 6 weeks after publication it was listed in the Google Play Store to be on the sixth rank of all medical Apps in Germany. Though this cannot be proven as well, these measures were interdependent and promoted FORTA as an integral package. The scientific papers were also essential, but are not likely to be often read by the main addressees of FORTA who are the GP. FORTA supports physicians (and clinical pharmacists) of all specialties, but only GP are able to integrate treatments across therapeutic areas as they have intricate knowledge on all major medical aspects of patients.

Therefore, we chose to place the survey in the setting of GP practices. Of course, no knowledge exists on the dissemination in other German states, or other countries where local FORTA lists have been established [14, 15].

This survey positively shows that dissemination of such instruments is possible, that FORTA seems to be implementable, teachable and useful as has already been shown in [7]. The final proof that the standard of medical care will be ameliorated by use of FORTA, however, still has to be provided by long-term trials under real-life conditions.

## Limitations

The survey has the typical limitations of all surveys: as the responder rate was low, a bias towards GP with knowledge on FORTA cannot be excluded. Thus, the positive findings reported here may be overestimating the true prevalence. Measures to increase responder rates (phone calls) may convey a more suggestive interrogation than the e-mail survey.

The qualitative and quantitative statements of GP were not confirmed by, for example, knowledge testing (CME tests), and may thus represent subjective overestimations by GP.

The development of FORTA was initiated and is still coordinated in the state of Baden-Württemberg; in other German states, or other countries, dissemination may be less advanced due to geographical distance.

## Conclusion

The survey shows that the clinically validated FORTA list and the related FORTA App have reached the level of their main addressees, the general practitioners, at least in one state of Germany. This dissemination success—though maybe overestimated due to the low response rate—also demonstrates the teachability, implementability and utility of these tools as the main prerequisites for the ultimate goal: the improvement in medical care for the rapidly increasing population of older people.
